# Novel monoclonal antibodies targeting the microtubule-binding domain of human tau

**DOI:** 10.1371/journal.pone.0195211

**Published:** 2018-04-02

**Authors:** Cara L. Croft, Brenda D. Moore, Yong Ran, Paramita Chakrabarty, Yona Levites, Todd E. Golde, Benoit I. Giasson

**Affiliations:** 1 Department of Neuroscience, College of Medicine, University of Florida, Gainesville, FL, United States of America; 2 Center for Translational Research in Neurodegenerative Disease, College of Medicine University of Florida, Gainesville, FL, United States of America; 3 McKnight Brain Institute, College of Medicine University of Florida, Gainesville, FL, United States of America; McGill University, CANADA

## Abstract

Tauopathies including Alzheimer’s disease and Progressive Supranuclear Palsy are a diverse group of progressive neurodegenerative disorders pathologically defined by inclusions containing aberrantly aggregated, post-translationally modified tau. The tau pathology burden correlates with neurodegeneration and dementia observed in these diseases. The microtubule binding domain of tau is essential for its physiological functions in promoting neuronal cytoskeletal stability, however it is also required for tau to assemble into an amyloid structure that comprises pathological inclusions. A series of novel monoclonal antibodies were generated which recognize the second and fourth microtubule-binding repeat domain of tau, thus enabling the identification specifically of 4-repeat tau versus 3-/4-repeat tau, respectively. These antibodies are highly specific for tau and recognize pathological tau inclusions in human tauopathies including Alzheimer’s disease and Progressive Supranuclear Palsy and in transgenic mouse models of tauopathies. These new antibodies will be useful for identifying and characterizing different tauopathies and as tools to target tau pathology in these diseases.

## Introduction

Tauopathies are a group of neurodegenerative diseases predominantly identifiable by inclusions composed of aggregated, highly phosphorylated and cleaved microtubule (MT)-associated protein tau (MAPT) [1]. This burden of tau inclusion pathology has been shown to correlate with the cognitive decline observed in these diseases, as well as, neurodegeneration [2–4]. Indeed, cognitive decline associates more with the tau burden compared to the amyloid-β load in Alzheimer’s disease (AD) [5]. Tauopathies are pathologically and symptomatically heterogeneous and include AD, Progressive Supranuclear Palsy (PSP), corticobasal degeneration (CBD), Pick’s disease, and frontotemporal dementia with parkinsonism linked to chromosome 17 (FTDP-17) [1, 6].

In brain, tau exists as six different isoforms, ranging from 352 to 441 amino acids in length as a result of alternative splicing of exons 2, 3 and 10 [7–9] and is natively unfolded [10]. Specifically, alternative splicing of exon 10 leads to the exclusion or inclusion of the second MT binding repeat (R2), producing tau isoforms with either 3 (3R) or 4 (4R) MT repeats, respectively [1, 6, 11]. Inclusion of exons 2 and 3 results in tau isoforms with N-terminal inserts termed N1 and N2, respectively [11, 12]. Tau expression is developmentally regulated; human fetal brain expresses only the shortest tau isoform (0N3R) whereas in healthy adult brain equal amounts of 3R and 4R tau are expressed [12, 13]. In disease, differences between 3R and 4R tau amounts can exist. Some FTDP-17 mutations increase the ratio of 4R to 3R tau and insoluble tau purified from these cases are largely 4R tau [1, 6, 13], similar to findings observed in PSP and CBD [14]. PiD, on the other hand shows increased 3R to 4R tau ratio [14] and in AD, equivalent amounts of both isoforms are found [15].

The MT binding domain (MTBD) of tau enables it to bind, assemble and stabilize MTs, promoting neuronal stability [1, 6, 11, 16–18]. The number of MT repeats (3R or 4R) in tau can affect the speed of axonal transport [19], and the presence of 4 MTBDs increases the propensity of tau to form β-sheets and aggregate [20]. The MTBD is the core domain required to drive amyloid structure formation and aggregation [21]. Phosphorylation in the MTBD decreases the affinity of tau for MTs [22] and may contribute to the accumulation of unbound tau in pathological inclusions.

Identifying 3R and 4R tau is relevant as pathological markers to distinguish between tauopathies and targeting the MT-binding repeat domain of tau is of interest for immunotherapy. Here we report the generation and characterization of a series of novel monoclonal antibodies targeting this region of tau enabling the study of these different isoforms and to therapeutically target tau pathology.

## Materials and methods

### Mice

All procedures were performed according to the NIH Guide for the Care and Use of Experimental Animals and were approved by the University of Florida Institutional Animal Care and Use Committee. Tau knockout (KO; tau -/-) mice [23] (Stock 007251) and tau transgenic (Tg) mice line PS19 expressing human 1N4R tau with the P301S mutation driven by the mouse prion promoter [24] were obtained from Jackson Laboratories (Stock 008169, Bar Harbor, ME, USA). JNPL3 tau transgenic mice expressing human 0N4R tau with the P301L mutation were previously described [25]. All mouse tissue for biochemical or immunohistochemical analysis were obtained from archival stocks, N = 10 per genotype. B6/C3H F1 mice (N = 10) (Envigo, Indianapolis, IN, USA) were used to generate antibodies.

### Production and purification of recombinant tau and α-synuclein proteins and recombinant tau fusion proteins

All recombinant proteins were expressed in *Escherichia coli* (*E*. *coli)* BL21 (DE3)/RIL (Agilent Technologies, Santa Clara, CA). Recombinant full-length human 2N3R and 2N4R tau, tau K18 fragment (corresponding to residues 244–372 relative to 2N4R human tau with an ATG codon added at the amino-terminus) and human α-synuclein (αS) were expressed using the respective cDNA cloned into the bacterial expression plasmid pRK172 and purified as previously described [13, 26]. All other chimeric protein cDNAs were generated by gene synthesis conducted by Genscript (Piscataway, NJ, USA) and cloned into pET16b vector. Recombinant 21–140 human αS with an ATG codon added at the amino-terminus followed by the nucleotide sequence for residues 244–372 in 4R human tau designated αS 21-140/K18 (used to generate antibodies 81A11 and 83E4) was purified using SP column with NaCl gradient elution flowed by size exclusion column. Recombinant 21–140 human αS with an ATG codon added at the amino-terminus followed by the nucleotide sequence for residues 244–372 in 4R human tau followed by human Aβ1–42 designated αS 21-140/K18/Aβ1–42 (94-3A2, 94-3A6, and 94-4F1 antibodies) was purified using using a HiTrap Q HP column (GE Healthcare Life Sciences) followed by size exclusion chromatography. Protein concentrations were determined by bicinchoninic acid (BCA) assay (Thermo Scientific) using bovine serum albumin (BSA) as the standard.

For the generation of recombinant glutathione S-transferases (GST) tagged human MTBD R1-3 (residues 244–336 relative to 2N4R tau), R1 (residues 244–274 relative to 2N4R tau), R3 (residues 306–336 relative to 2N4R tau) and R4 (residues 337–372 relative to 2N4R tau) proteins, the corresponding cDNA sequences were amplified by PCR with the respective oligonucleotides with added BamHI and EcoRI restriction sites and cloned in these sites of bacterial expression plasmid pGEX-2T. These proteins were expressed in *Escherichia coli* (*E*. *coli)* BL21 (DE3)/RIL with isopropyl β-D-1-thiogalactopyranoside induction. The bacteria were lysed with 2% SDS and following the addition of SDS sample buffer equal amounts of total bacterial lysates were resolved by SDS-PAGE and analyzed by immunoblotting.

### Generation of new tau mouse monoclonal antibodies

Female B6/C3H F1 mice (N = 5 per peptide) were used for immunization with synthetic peptides corresponding to the MTBD in 2N4R human tau 244–372 (K18) conjugated to 21–140 αS at the N-terminus (αS 21-140/K18) or 21–140 αS at the N-terminus plus Aβ1–42 at the C-terminus (αS 21-140/K18/Aβ1–42). Protein (100 μg) in 200 μl phosphate buffered saline (PBS) were emulsified with either 100 μl of Freunds complete adjuvant (1^st^ injection; Sigma Aldrich, St. Louis, MO) or Freunds incomplete adjuvant (subsequent injections; Sigma Aldrich, St. Louis, MO). For the first immunization, mice were injected subcutaneously. An intraperitoneal (IP) injection was administered 3 weeks later. Six weeks following the initial injection, mice were boosted with an IP injection of the proteins in PBS. Three days later, mice were euthanized by CO_2_ inhalation and spleens were harvested using aseptic technique.

Mouse myeloma (Sp2/O-Ag14; ATCC, Manassas, VA) cells were maintained in high glucose (4.5gm/L) Dulbecco’s Modified Eagle Medium (DMEM) with 10% NCTC 135 Media (Sigma Aldrich, St. Louis, MO), 20% hybridoma grade fetal bovine serum (FBS; Hyclone, Logan, UT), 100 U/ml penicillin, 100 U/ml streptomycin, 2 mM L-glutamine, 0.45 mM pyruvate, 1 mM oxaloacetate, and 0.2 U/ml insulin at 37°C and 8% CO_2_. Spleens were gently homogenized in 5% FBS/Hank’s balanced salt solution (HBSS; Lonza, Walkersville, MD). Cell suspensions were collected and centrifuged to pellet cells. The cell pellets were resuspended in red blood cell lysis buffer (Sigma Aldrich, St. Louis, MO) and diluted with HBSS after one minute. The cells were then washed twice by centrifuging at 100 x g for 10 minutes and resuspending in HBSS. Sp2/O-Ag14 cells were also washed twice with HBSS. Five million Sp2/O-Ag14 cells were added to 50 million spleen cells and, after centrifuging at 100 x g for 10 minutes onto a culture dish, fusion was induced with 50% polyethylene glycol 1450 (Sigma Aldrich, St. Louis, MO). After washing with HBSS, cells were incubated in Sp2/O-Ag14 media at 37°C with 8% CO_2_ overnight. The next day, the cells were gently detached from the plate and distributed into 96 well plates with Sp2/O-Ag14 media/0.5% hybridoma enhancing supplement (Sigma Aldrich, St. Louis, MO)/HAT selection supplement (Sigma Aldrich, St. Louis, MO).

### Hybridoma screening

All hybridoma clones were screened for reactivity to K18 versus αS and Aβ by enzyme-linked immunosorbent assay (ELISA). MaxiSorp plates (Thermo Scientific, Waltham, MA) or Immulon 4HBX plates (ThermoFisher Scientific, Waltham, MA) were coated with 1 μg/ml human recombinant tau, αS or Aβ in PBS or 100 mM sodium bicarbonate and blocked with 5% FBS/PBS or 1% Block ACE in PBS. Media from the hybridomas were applied to plates, which were then incubated at room temperature for 3 hours. Next, the plates were washed with PBS, and incubated with goat anti-mouse secondary antibody conjugated to horse radish peroxidase (HRP; Jackson Immuno Research Labs, West Grove, PA) for 1 hour at room temperature. Then, plates were washed and TMB substrates (Pierce, Rockford, IL) were applied until color changes were observed. Reactions were then quenched with 1M HCl or 85% O-Phosphoric acid and absorbance was measured at 450 nm. Clones that were positive by ELISA were transferred to larger culture plates as needed.

Antibody clones were isotyped with the mouse monoclonal antibody isotyping kit purchased from Sigma-Aldrich (St. Louis, MO).

### Preparation of total mouse brain protein lysates

Tau KO, PS19 Tg or non-transgenic (NTg) mice were humanely euthanized by CO_2_ inhalation and the brains were harvested. Brain tissue was homogenized in 2% SDS/50 mM Tris, pH 7.5 using a probe sonicator and then incubated for 10 min at 100°C. Protein concentrations were determined by BCA assay using BSA as the standard. Sample buffer was added and equal amounts of protein (10 μg) were resolved by SDS-PAGE and analyzed by immunoblot.

### Preparation of sarkosyl-insoluble human temporal cortex

All frozen human brain tissue was obtained from the University of Florida Neuromedicine Human Brain Tissue Bank. All cases were de-identified before authors obtained the tissue. Pulverized temporal cortex tissue from human control (n = 2) or AD cases (n = 3) was homogenized in high-salt (HS) buffer (50 mM Tris–HCl, pH 7.5, 0.75 M NaCl, 2 mM EDTA, 50 mM NaF with protease inhibitor cocktail [Roche]) at 3 ml buffer/g tissue and centrifuged at 100,000 x g for 30 minutes at 4°C. Supernatants were collected (HS fraction) and pellets were resuspended in HS buffer containing 1% Triton X-100 at 2 ml buffer/g tissue. Samples were centrifuged at 100,000 x g for 30 minutes at 4°C and the supernatants were collected (HS/Triton-soluble fraction). Pellets were washed in the same buffer and then re-suspended in HS buffer containing 1% sarkosyl at 1 ml buffer/g tissue and incubated at 37°C for 30 minutes, and centrifuged at 100,000 x g for 30 minutes at 4°C and supernatants were collected (sarkosyl-soluble fraction). The detergent-insoluble pellets were extracted in 0.5 ml of 4 M urea, 2% SDS, 25 mM Tris–HCl pH 7.6/g tissue, sonicated, and sedimented at 100,000 x g for 30 minutes at 25°C. Protein concentrations were determined by BCA assay (Thermo Scientific) using BSA as the standard. SDS sample buffer was added, and equal amounts of protein (10 μg) were resolved by SDS-PAGE and analyzed by immunoblot.

### Immunoblotting

Protein samples were resolved by electrophoresis on 4–12% Bis‐Tris precast gels (Biorad, Hercules, CA, USA), then electrophoretically transferred to polyvinylidene fluoride (PVDF) membranes. Membranes were blocked with 0.5% casein in Tris-buffered saline (TBS) then incubated overnight at 4°C with primary antibodies diluted in 0.5% casein in TBS. A goat anti-GST antibody was obtained from GE Healthcare Biosciences (Pittsburgh, PA, USA). Following washing, blots were incubated with fluorophore-conjugated secondary antibodies diluted in 0.5% casein in TBS for 1 hour. Following washing, protein bands were visualized and quantified using an Odyssey infrared imaging and analysis system (Li-Cor Biosciences, Lincoln, NE, USA).

### Immunohistochemistry

Paraffin embedded tissue from JNPL3 tau transgenic [25], PS19 tau-transgenic [24] and NTg mice was used. All paraffin embedded, formalin fixed human brain tissue from de-identified donors was obtained through the University of Florida Neuromedicine Human Brain Tissue Bank. Sequential tissue sections were deparaffinized with xylenes, and sequentially rehydrated with graded ethanol solutions (100–70%). Antigen retrieval was performed by incubating sections in a steam bath for 30 minutes. Endogenous peroxidase activity was quenched with 1.5% hydrogen peroxide/0.005% Triton-X-100/TBS for 20 minutes. Sections were blocked with 2.5% horse serum then incubated with primary antibody overnight at 4°C. Following washing with TBS, sections were incubated with Vector ImmPress anti-mouse IgG peroxidase (Vector Laboratories, Burlingame, CA, USA) for 30 minutes. Sections were washed with TBS and then developed with 3, 3’diaminobenzidine (DAB kit; Vector Laboratories). Reactions were stopped by immersing the slides in TBS and sections were counterstained with Mayer's hematoxylin (Sigma Aldrich, St. Louis, MO). Next, sections were dehydrated with an ascending series of ethanol solutions (70%-100%) followed by xylenes, and coverslipped using cytoseal (Thermo Scientific, Waltham, MA).

## Results

### Generation of antibodies targeting the microtubule binding domain of tau

**Fig 1:**
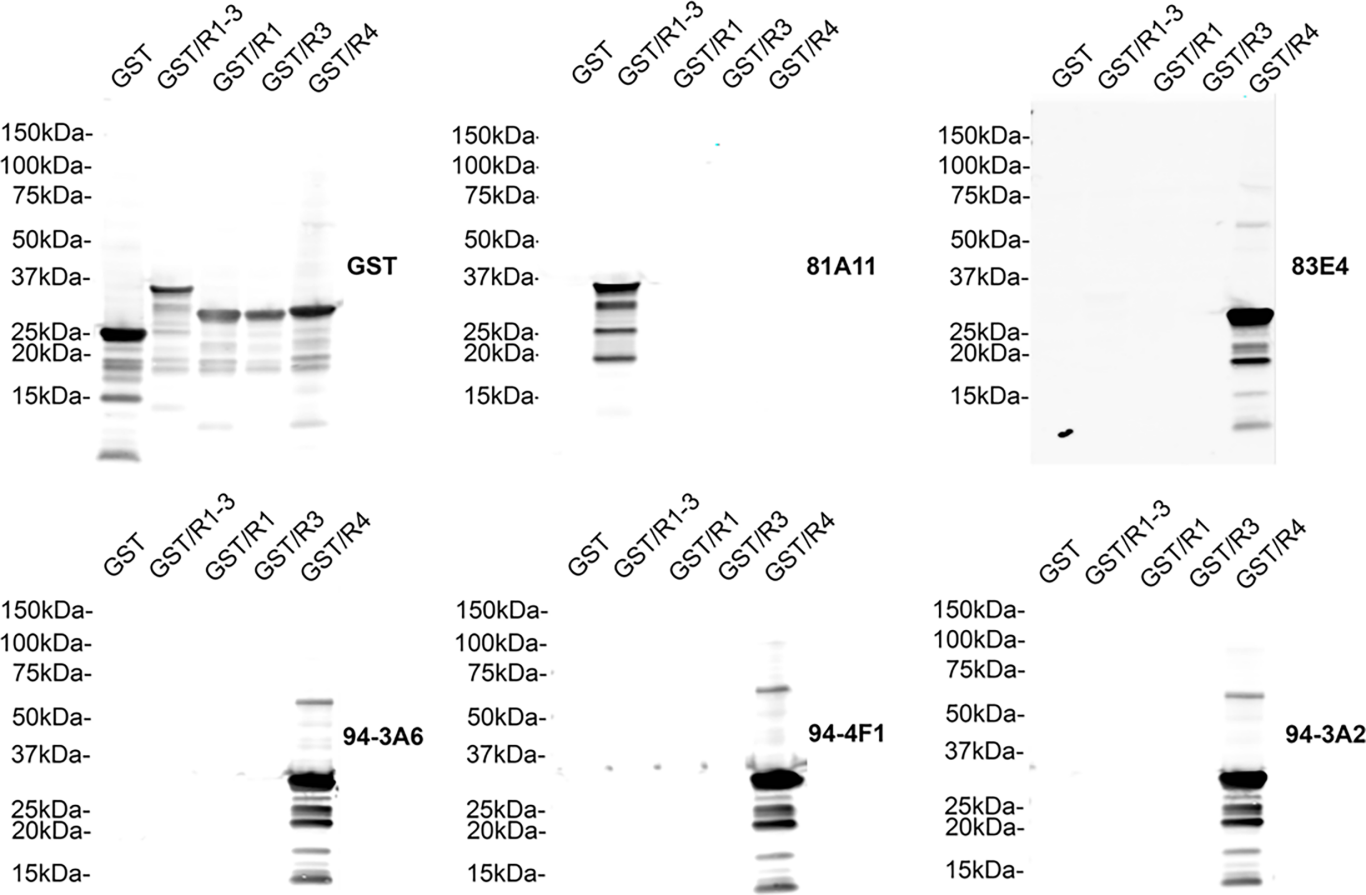
Specificity of novel tau antibodies to the MT-binding repeat domains of tau. Immunoblots of recombinant glutathione-S-transferase (GST), GST/R1-3, GST/R1, GST/R3 and GST/R4 human tau probed with anti-GST antibody and the novel tau antibodies (as indicated for each blot) 81A11, 83E4, 94-3A2, 94-3A6, 94-4F1 to determine epitopes. The mobilities of molecular mass markers are shown on the left.

**Fig 2:**
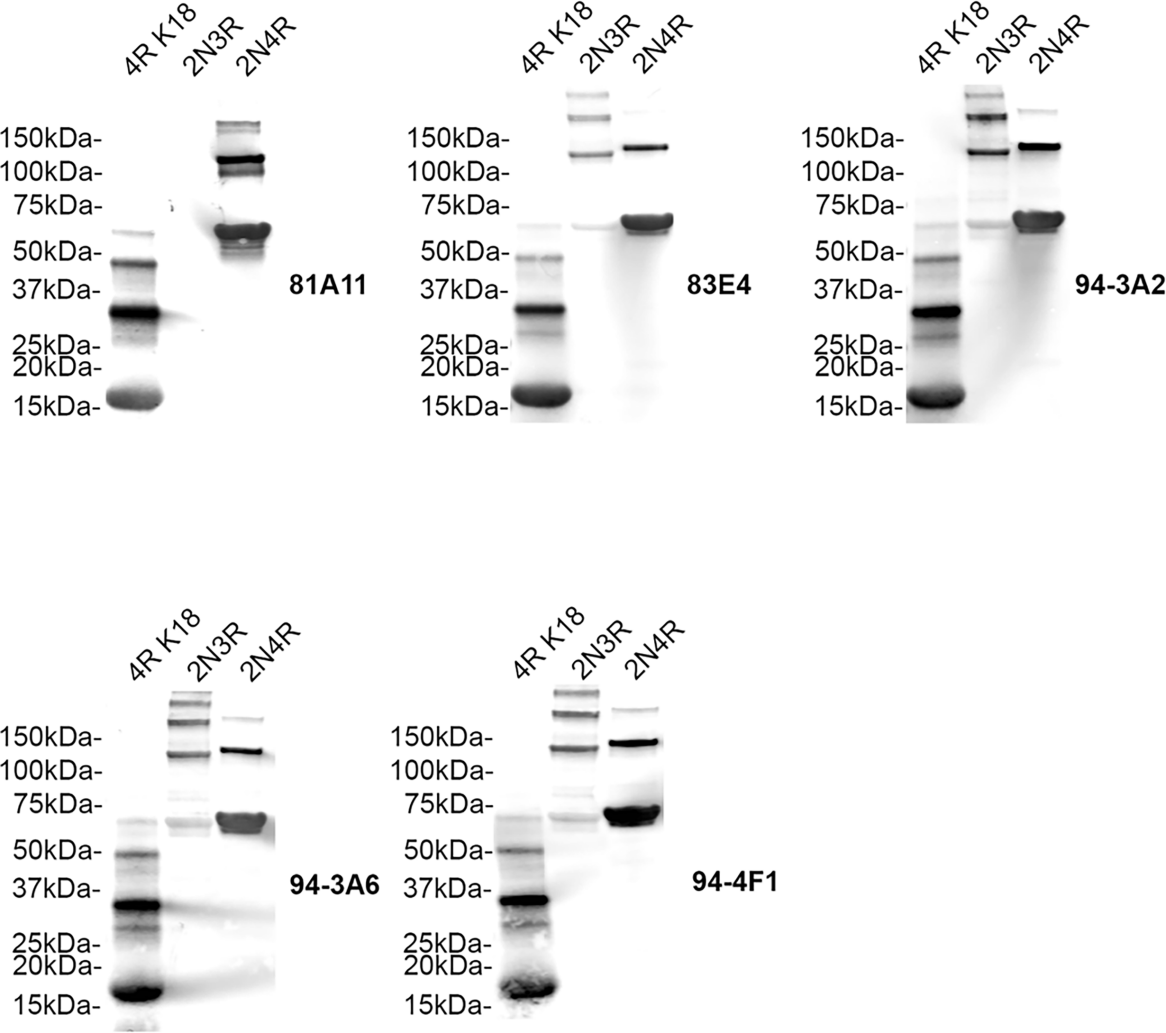
Specificity of novel tau antibodies to recombinant 3-repeat and 4-repeat human tau proteins. Immunoblots of recombinant K18 tau fragment (4R MTBD) and full-length human 2N3R and 2N4R tau were probed with the novel tau antibodies (as indicated for each blot) 81A11, 83E4, 94-3A2, 94-3A6, 94-4F1 to determine specificity for 3R and 4R tau. The mobilities of molecular mass markers are shown on the left.

Mice were immunized with the synthetic peptides corresponding to the MTBD in 4R human tau 244–372 (K18) conjugated to 21–140 αS at the N-terminus or 21–140 αS at the N-terminus plus Aβ1–42 at the C-terminus. These fusion proteins were used to enhance the immunogenicity of the peptide and increase the likelihood of obtaining suitable tau antibodies, particularly as αS is a highly immunogenic soluble protein. Several hybridomas were identified by ELISA screening that showed reactivity to recombinant human tau but not to recombinant human αS or Aβ. This selectivity for tau was also confirmed by detection of tau pathology by immunohistochemistry of human AD post-mortem brain tissue with abundant tau pathology. Five hybridomas (81A11, 83E4, 94-3A2, 94-3A6, 94-4F1) were identified using these criteria. To map the epitopes of these monoclonal antibodies, immunoblots of recombinant GST tagged human MTBD R1-3, R1, R3 and R4 were probed with these antibodies (Fig 1). 81A11 is the only antibody to recognize R1-3 but not R1 or R3 alone highlighting its selectivity for R2, and the remaining antibodies (83E4, 94-3A2, 94-3A6, 94-4F1) selectively bind R4 only. We further determined the specificity of these monoclonal antibodies by probing immunoblots of recombinant full-length human 2N3R and 2N4R tau and K18 (4R) tau fragment [7, 27] (Fig 2). 81A11 recognizes both 2N4R tau and K18 but not 2N3R tau highlighting its specificity for R2 within the MTBD of tau. The remaining antibodies 83E4, 94-3A2, 94-3A6, 94-4F1 recognize both 3R and 4R tau due to their selectivity for R4 within the MTBD of tau.

### Characterization of antibodies with tau transgenic mouse tissue

**Fig 3:**
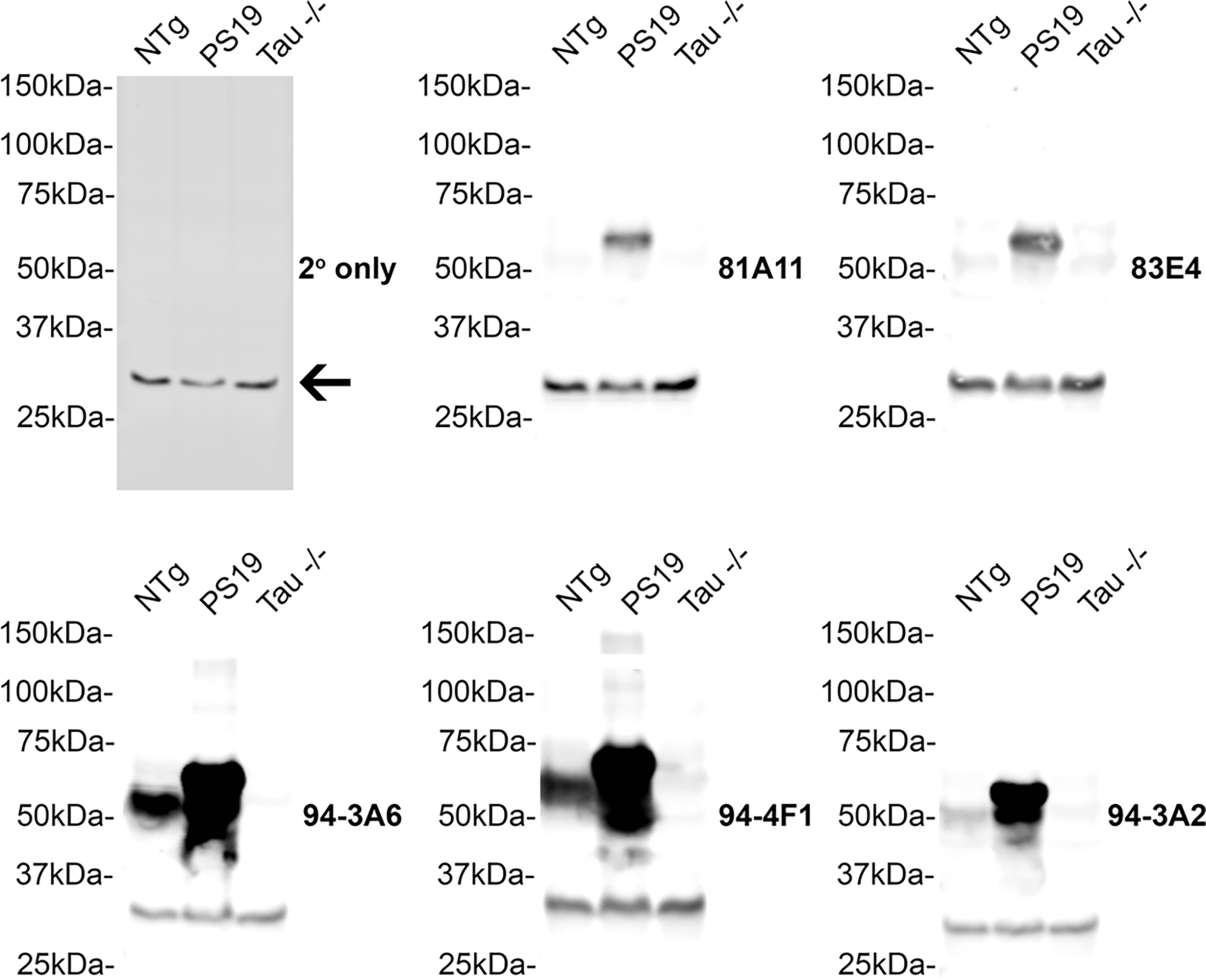
Characterization of novel tau antibodies in NTg, tau KO and PS19 tau Tg mice. Immunoblots of total brain lysates from NTg, PS19 tau transgenic and tau KO (-/-) mice were probed with the novel tau antibodies (as indicated for each blot) 81A11, 83E4, 94-3A2, 94-3A6, 94-4F1 to determine affinity for tau in mice. An immunoblot probed with secondary antibody only highlights a non-specific band present on all immunoblots (arrow). The mobilities of molecular mass markers are shown on the left.

To further determine the selectivity of this series of antibodies for human tau, immunoblots of total mouse brain lysates from tau KO [23], NTg and PS19 tau Tg mice [24] were probed with these antibodies (Fig 3). All of the new tau antibodies detected human tau in whole brain lysates from PS19 mice. Antibodies 81A11, 83E4, and 94-3A2 reacted weakly with endogenous tau in NTg mice. Antibodies 94-3A6 and 94-4F1 demonstrated stronger reactivity for endogenous tau in NTg mice. All the antibodies were specific for tau as shown using the lysates from tau KO mice. A non-specific band at approximately 30 kDa present in all the lysates was attributed to non-specific binding of the secondary antibody shown with an immunoblot probed only with secondary antibody.

**Fig 4:**
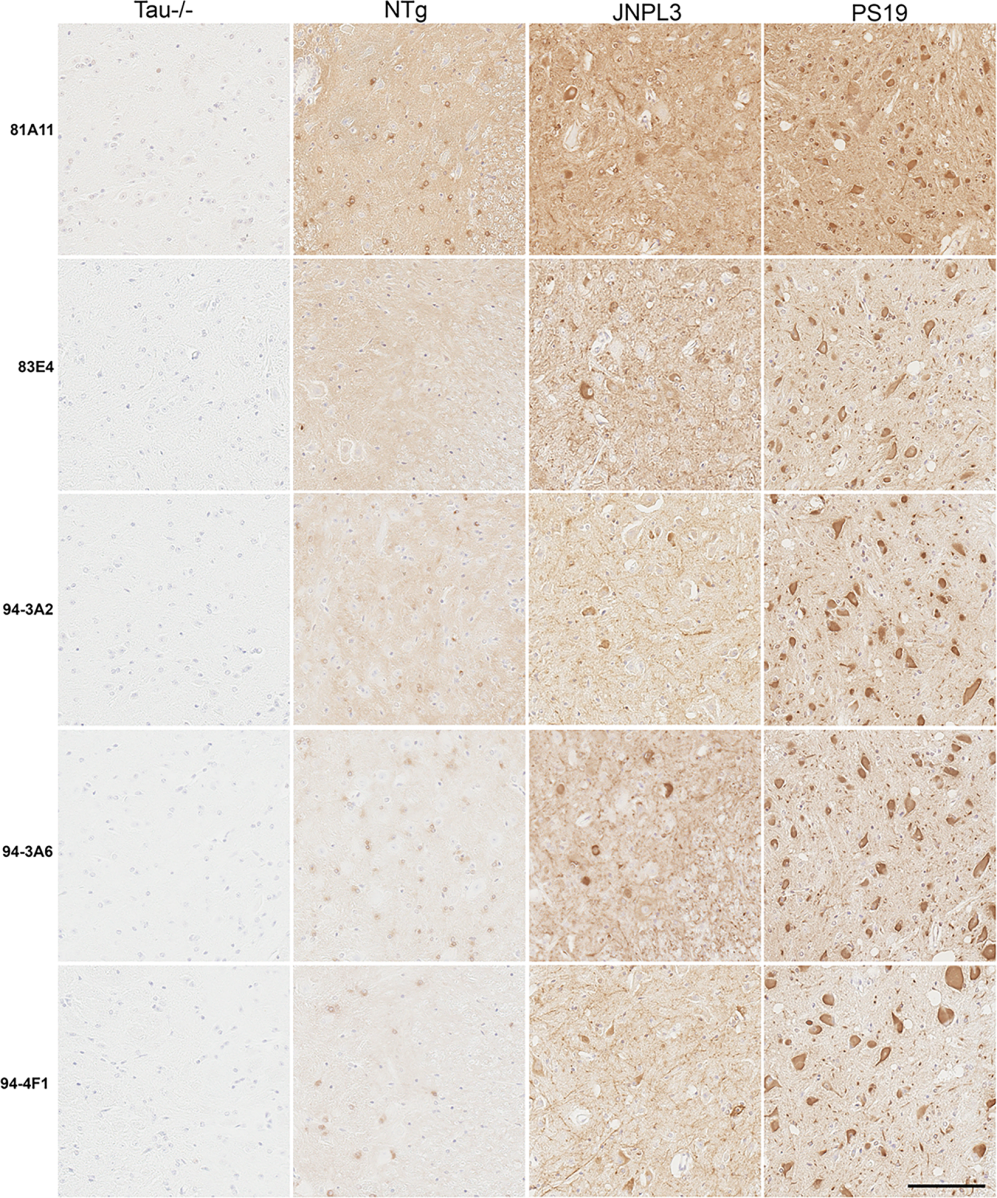
Immunohistological identification of tau inclusions in JNPL3 tau Tg mice and PS19 tau Tg mice. Immunohistochemistry of representative tau pathology in the spinal cord of 7.5 month old JNPL3 Tg mice and 13 month old PS19 Tg mice detected with the novel tau antibodies 81A11, 83E4, 94-3A2, 94-3A6, 94-4F1. A 7 month old NTg mouse and 6 month old tau KO (Tau-/-) mouse are also shown. Scale bar = 100 μm.

The ability of these new antibodies to react with tau inclusions in the JNPL3 [25] and PS19 mouse model of tauopathy was also examined by immunohistochemistry (Fig 4). This panel of antibodies detects tau inclusions in the spinal cord of JNPL3 and PS19 mice and detects endogenous tau in an age-matched NTg spinal cord. No reactivity is observed in spinal cord from a tau KO mouse.

### Characterization of antibodies with human post-mortem AD and PSP tissue

**Fig 5:**
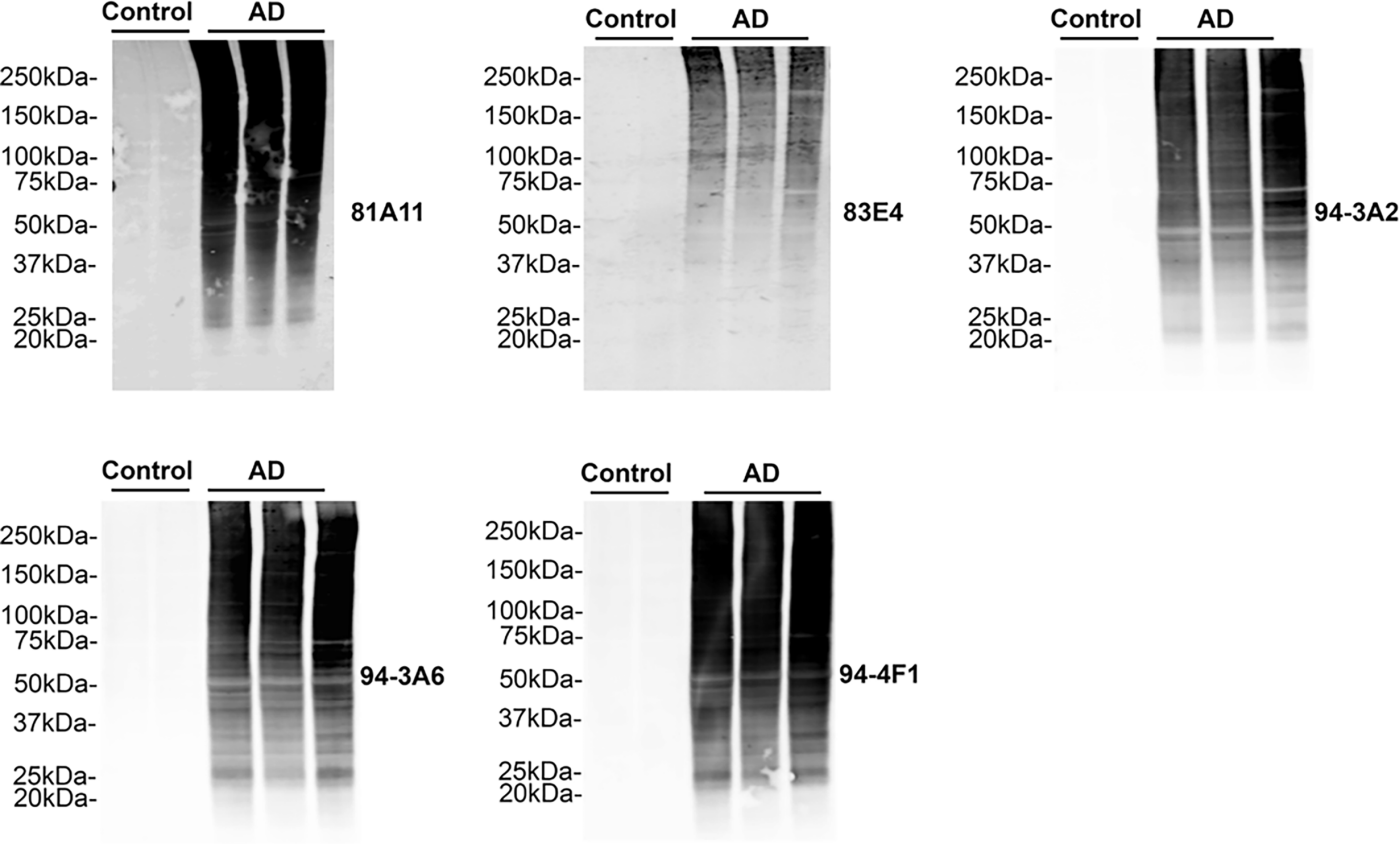
Characterization of novel tau antibodies in sarkosyl-insoluble human control and AD cortex. Immunoblots of biochemically sarkosyl-insoluble temporal cortex lysates from post-mortem control (n = 2) and AD (n = 3) cases were probed with the novel tau antibodies (as indicated for each blot) 81A11, 83E4, 94-3A2, 94-3A6, 94-4F1 to determine affinity for tau in human AD. The mobilities of molecular mass markers are shown on the left.

To further characterize these novel monoclonal antibodies, sarkosyl-insoluble tau prepared from sequential extractions of temporal cortex tissue from AD and control cases was also assessed by immunoblotting (Fig 5). All antibodies specifically reacted with sarkosyl-insoluble tau in the AD cases (N = 3), showing no reactivity in samples from control cases (N = 2). The aggregated and post-translationally modified tau in the AD cases was detected with all these antibodies as proteins smears from below the expected molecular mass of naïve tau to the top of gels.

**Fig 6:**
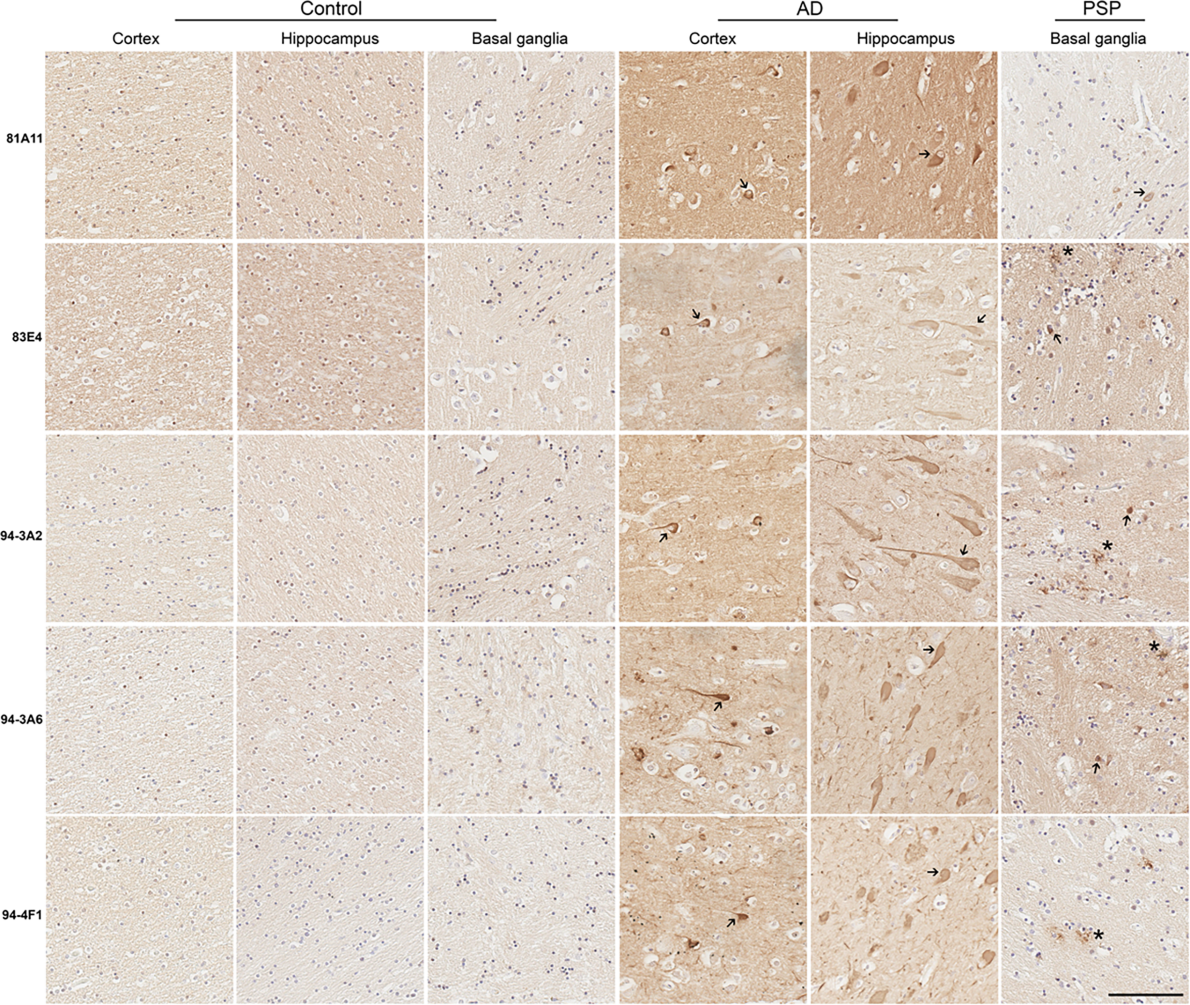
Immunohistological reactivity of tau inclusions in human post-mortem AD cortex and hippocampus and PSP basal ganglia. Immunohistochemistry of representative tau pathology in post-mortem AD and control temporal cortex and hippocampus and in PSP and control basal ganglia detected with the novel tau antibodies 81A11, 83E4, 94-3A2, 94-3A6, 94-4F1. Asterisks mark astroglial tau inclusions and arrows mark NFTs. Scale bar = 100 μm.

Neurofibrillary tangles (NFTs) are predominantly found in AD hippocampus and cortex upon histological examination [28, 29]; therefore, the ability of these antibodies to detect NFTs in AD brain sections was examined by immunohistochemistry (Fig 6). All of these antibodies reacted with NFTs in AD cortex and hippocampus. In PSP, tau inclusions also form in the glia of the basal ganglia and brain stem, as well as, the development of some globose NFTs [30, 31]. The ability of these antibodies to detect tau inclusions in PSP basal ganglia was examined by immunohistochemistry (Fig 6). 83E4, 94-3A2, 94-3A6, and 94-4F1 highlighted glial tau inclusions and some globose NFTs in PSP sections. 81A11 only weakly detected the tau inclusions in PSP tissue.

## Discussion

Here, we present data where novel mouse monoclonal antibodies which recognize epitopes within the MTBD of tau were generated. We have validated that these antibodies are highly-specific for tau and show reactivity for pathological inclusions in tauopathy mice and human AD and PSP. A summary of these new monoclonal tau antibodies is shown in Table 1.

**Table 1:** Novel MTBD tau antibodies generated in this study. Summary of the new antibodies generated and characterized in this study, including the antigens used to generate each antibody, the specificity for each antibody, and their isotypes.

Antibody	Antigen	Specificity	Isotype
81A11	αS 21-140/K18	R2	IgG1
83E4	αS 21-140/K18	R4	IgG1
94-3A2	αS 21-140/K18/Aβ1–42	R4	IgG2a
94-3A6	αS 21-140/K18/Aβ1–42	R4	IgG2b
94-4F1	αS 21-140/K18/Aβ1–42	R4	IgG2a

In the first instance, we determined that 81A11 reacts with the R2 repeat and shows selectivity for 4-repeat tau and the remaining antibodies in the series (83E4, 94-3A2, 94-3A6, 94-4F1) bind R4 and can recognize 3- and 4-repeat tau. We then examined reactivity with mouse brain tissue by immunohistochemistry and immunoblotting; all of these monoclonal antibodies show some reactivity to endogenous mouse tau in the NTg tissue, as 0N4R is the main isoform of tau expressed in adult mice [32] as well as strongly recognizing the over-expressed 1N4R human tau in PS19 brain [24]. These antibodies show no cross-reactivity with tau -/- brain demonstrating the high selectivity of these antibodies to tau. In addition, this panel of antibodies recognize NFTs in the spinal cord of JNPL3 and PS19 mice highlighting their utility to recognize inclusion pathology in these models.

Furthermore, this panel of antibodies can robustly identify tau from sarkosyl-insoluble preparations of AD cortical brain tissue by immunoblotting, showing no reactivity with the same lysates prepared from human control cortex. These antibodies also recognize pathological tau inclusions upon immunohistochemical examination; NFTs and glial and globose NFT inclusions in AD and PSP, respectively, were recognized by these antibodies in regions of pathological interest. Endogenous staining of tau is also observed in control cases. Of note, 81A11 which recognizes R2 of the MTBD of tau does not recognize pathological tau inclusions in PSP brain as well as the other antibodies which recognize R4 and it could be speculated that this epitope is masked due to the amyloidogenic β-sheets forming in this region of tau [21, 33]. Other antigen retrieval methods not employed in this study could also increase the ability of 81A11 to recognize 4-repeat tau in the PSP cases [34].

The MTBD of tau is of therapeutic interest because it is in this region where tau forms its amyloidogenic structure [20, 21, 33]. Indeed, in tau, β-sheet structures flanked by the second (275–280 amino acids) and the third (306–311 amino acids) repeats of MTBD enable the formation of paired and straight helical filaments which are characteristic of tauopathies such as AD and PSP [33]. It is also this region of tau where post-translational modifications permit the disassembly of microtubules, thereby increasing amounts of unbound tau, which can accumulate in the pathological brain [1, 35].

At present, there are no effective therapeutic strategies available to target tauopathies [36]. There have been numerous failures in anti-Aβ therapies for AD [37], and the correlation between cognitive decline and tau pathology in human AD [5] has driven interest to target tau pathology in AD. Some of these therapies may also be useful for other tauopathies. In particular, there is now a body of evidence implicating the propagation and release of tau as a disease mechanism in AD and other tauopathies [38–41]; suggesting immunotherapy intervention with antibodies could prevent the propagation of such pathology. Indeed, several tau immunotherapies have been employed pre-clinically and have shown efficacy in these pre-clinical models [42–45]. Furthermore, the humanized tau antibody (ABBV-8E12) has received Phase 2 approval for early AD and PSP (Clinical Trial #NCT02880956 and #NCT02985879). Additionally, recent evidence has suggested that balancing 3R and 4R tau isoforms is effective in pre-clinical studies [46] and antibodies specific to 3R or 4R tau could enable the rebalancing of tau isoforms.

Tauopathies encompass a diverse spectrum of phenotypes and pathologies. There is much interest in immunotherapy against tauopathies, but at this stage very little is known as to which antibody strategy may be the most appropriate, and it is likely this will differ between different tauopathies, as well as, disease stage. The novel tau antibodies to the MTBD of tau described here will enable exploration of targeting this region of tau therapeutically in future work.

## Conclusions

We have generated and characterized a series of highly tau-specific novel monoclonal antibodies, which recognize the MTBD of tau. Importantly, one of these antibodies specifically targets the R2 repeat domain, enabling the identification of 4-repeat tau. These monoclonal antibodies detect pathological inclusions in transgenic tau mice and in human tauopathy brain. These tau MTBD antibodies will be useful to examine tau pathology and as promising novel immunotherapies.

## References

[1] Guo T, Noble W, Hanger DP. Roles of tau protein in health and disease. Acta Neuropathol. 2017;133(5):665–704. doi: 10.1007/s00401-017-1707-9 28386764 10.1007/s00401-017-1707-9 PMC5390006

[2] Augustinack JC, Schneider A, Mandelkow EM, Hyman BT. Specific tau phosphorylation sites correlate with severity of neuronal cytopathology in Alzheimer's disease. Acta Neuropathol. 2002;103(1):26–35. 11837744 10.1007/s004010100423

[3] Gomez-Isla T, Hollister R, West H, Mui S, Growdon JH, Petersen RC, et al Neuronal loss correlates with but exceeds neurofibrillary tangles in Alzheimer's disease. Ann Neurol. 1997;41(1):17–24. doi: 10.1002/ana.410410106 9005861 10.1002/ana.410410106

[4] Perez-Nievas BG, Stein TD, Tai H-C, Dols-Icardo O, Scotton TC, Barroeta-Espar I, et al Dissecting phenotypic traits linked to human resilience to Alzheimer’s pathology. Brain. 2013;136(8):2510–26. 23824488 10.1093/brain/awt171 PMC3722351

[5] Brier MR, Gordon B, Friedrichsen K, McCarthy J, Stern A, Christensen J, et al Tau and Aβ imaging, CSF measures, and cognition in Alzheimer’s disease. Sci Transl Med 2016;8(338):338ra66–ra66. doi: 10.1126/scitranslmed.aaf2362 27169802 10.1126/scitranslmed.aaf2362 PMC5267531

[6] Wang Y, Mandelkow E. Tau in physiology and pathology. Nat Rev Neurosci. 2016;17(1):22–35. doi: 10.1038/nrn.2015.3 26631930 10.1038/nrn.2015.1

[7] Goedert M, Spillantini MG, Jakes R, Rutherford D, Crowther RA. Multiple isoforms of human microtubule-associated protein tau: sequences and localization in neurofibrillary tangles of Alzheimer's disease. Neuron. 1989;3(4):519–26. 2484340 10.1016/0896-6273(89)90210-9

[8] Himmler A. Structure of the bovine tau gene: alternatively spliced transcripts generate a protein family. Mol Cell Biol 1989;9(4):1389–96. 2498650 10.1128/mcb.9.4.1389-1396.1989 PMC362555

[9] Goedert M, Spillantini MG, Potier MC, Ulrich J, Crowther RA. Cloning and sequencing of the cDNA encoding an isoform of microtubule-associated protein tau containing four tandem repeats: differential expression of tau protein mRNAs in human brain. EMBO J. 1989;8(2):393–9. 2498079 10.1002/j.1460-2075.1989.tb03390.x PMC400819

[10] Schweers O, Schönbrunn-Hanebeck E, Marx A, Mandelkow E. Structural studies of tau protein and Alzheimer paired helical filaments show no evidence for beta-structure. J Biol Chem. 1994;269(39):24290–7. 7929085

[11] Himmler A, Drechsel D, Kirschner MW, Martin DW. Tau consists of a set of proteins with repeated C-terminal microtubule-binding domains and variable N-terminal domains. Mol Cell Biol. 1989;9(4):1381–8. 2498649 10.1128/mcb.9.4.1381-1388.1989 PMC362554

[12] Goedert M, Jakes R. Expression of separate isoforms of human tau protein: correlation with the tau pattern in brain and effects on tubulin polymerization. EMBO J. 1990;9(13):4225–30. 2124967 10.1002/j.1460-2075.1990.tb07870.x PMC552204

[13] Hong M, Zhukareva V, Vogelsberg-Ragaglia V, Wszolek Z, Reed L, Miller BI, et al Mutation-specific functional impairments in distinct Tau isoforms of hereditary FTDP-17. Science. 1998;282(5395):1914–7. 9836646 10.1126/science.282.5395.1914

[14] Buée L, Delacourte A. Comparative biochemistry of Tau in progressive supranuclear palsy, corticobasal degeneration, FTDP-17 and Pick's disease. Brain Pathol. 1999;9(4):681–93. 10517507 10.1111/j.1750-3639.1999.tb00550.x PMC8098140

[15] Espinoza M, de Silva R, Dickson DW, Davies P. Differential incorporation of Tau isoforms in Alzheimer’s disease. J Alzheimer's dis. 2008;14(1):1–16. 18525123 10.3233/jad-2008-14101 PMC2882247

[16] Noble W, Hanger DP, Miller CCJ, Lovestone S. The importance of Tau phosphorylation for neurodegenerative diseases. Front Neurol. 2013;4:83 doi: 10.3389/fneur.2013.00083 23847585 10.3389/fneur.2013.00083 PMC3696910

[17] Weingarten MD, Lockwood AH, Hwo SY, Kirschner MW. A protein factor essential for microtubule assembly. Proc Natl Acad Sci USA. 1975;72(5):1858–62. 1057175 10.1073/pnas.72.5.1858 PMC432646

[18] Brandt R, Lee G. Orientation, assembly, and stability of microtubule bundles induced by a fragment of tau protein. Cell Motil Cytoskeleton. 1994;28(2):143–54. doi: 10.1002/cm.970280206 8087873 10.1002/cm.970280206

[19] Peck A, Sargin ME, LaPointe NE, Rose K, Manjunath BS, Feinstein SC, et al Tau isoform-specific modulation of kinesin-driven microtubule gliding rates and trajectories as determined with tau-stabilized microtubules. Cytoskeleton. 2011;68(1):44–55. doi: 10.1002/cm.20494 21162159 10.1002/cm.20494

[20] Mukrasch MD, Bibow S, Korukottu J, Jeganathan S, Biernat J, Griesinger C, et al Structural polymorphism of 441-residue Tau at single residue resolution. PLoS Biol. 2009;7(2):e1000034. 10.1371/journal.pbio.1000034 PMC2642882 19226187

[21] Fitzpatrick AWP, Falcon B, He S, Murzin AG, Murshudov G, Garringer HJ, et al Cryo-EM structures of tau filaments from Alzheimer’s disease. Nature. 2017;547(7662):185–90. doi: 10.1038/nature23002 28678775 10.1038/nature23002 PMC5552202

[22] Wang J-Z, Gong C-X, Zaidi T, Grundke-Iqbal I, Iqbal K. Dephosphorylation of Alzheimer paired helical filaments by protein phosphatase-2A and −2B. J Biol Chem. 1995;270(9):4854–60. 7876258 10.1074/jbc.270.9.4854

[23] Dawson HN, Ferreira A, Eyster MV, Ghoshal N, Binder LI, Vitek MP. Inhibition of neuronal maturation in primary hippocampal neurons from τ deficient mice. J Cell Sci. 2001;114(6):1179–87. 11228161 10.1242/jcs.114.6.1179

[24] Yoshiyama Y, Higuchi M, Zhang B, Huang S-M, Iwata N, Saido Takaomi C, et al Synapse loss and microglial activation precede tangles in a P301S tauopathy mouse model. Neuron. 2007;53(3):337–51. doi: 10.1016/j.neuron.2007.01.010 17270732 10.1016/j.neuron.2007.01.010

[25] Lewis J, McGowan E, Rockwood J, Melrose H, Nacharaju P, Van Slegtenhorst M, et al Neurofibrillary tangles, amyotrophy and progressive motor disturbance in mice expressing mutant (P301L) tau protein. Nature Genet. 2000;25(4):402–5. doi: 10.1038/78078 10932182 10.1038/78078

[26] Giasson BI, Forman MS, Higuchi M, Golbe LI, Graves CL, Kotzbauer PT, et al Initiation and synergistic fibrillization of Tau and alpha-synuclein. Science. 2003;300(5619):636–40. doi: 10.1126/science.1082324 12714745 10.1126/science.1082324

[27] Trinczek B, Biernat J, Baumann K, Mandelkow EM, Mandelkow E. Domains of tau protein, differential phosphorylation, and dynamic instability of microtubules. Mol Biol Cell. 1995;6(12):1887–902. 8590813 10.1091/mbc.6.12.1887 PMC366657

[28] Braak H, Braak E. Neuropathological stageing of Alzheimer-related changes. Acta Neuropathol. 1991;82(4):239–59. 1759558 10.1007/BF00308809

[29] Braak H, Braak E. Staging of alzheimer's disease-related neurofibrillary changes. Neurobiol Aging. 1995;16(3):271–8. 7566337 10.1016/0197-4580(95)00021-6

[30] Komori T. Tau-positive dial Inclusions in progressive supranuclear palsy, corticobasal degeneration and Pick's disease. Brain Pathol. 1999;9(4):663–79. 10517506 10.1111/j.1750-3639.1999.tb00549.x PMC8098509

[31] Steele JC, Richardson J, Olszewski J. Progressive supranuclear palsy: A heterogeneous degeneration involving the brain stem, basal ganglia and cerebellum with vertical gaze and pseudobulbar palsy, nuchal dystonia and dementia. Arch Neurol. 1964;10(4):333–59. 14107684 10.1001/archneur.1964.00460160003001

[32] McMillan P, Korvatska E, Poorkaj P, Evstafjeva Z, Robinson L, Greenup L, et al Tau isoform regulation is region and cell-specific in mouse brain. J Comp Neurol. 2008;511(6):788–803. doi: 10.1002/cne.21867 18925637 10.1002/cne.21867 PMC2845852

[33] Mandelkow E, Von Bergen M, Biernat J, Mandelkow E-M. Structural principles of Tau and the paired helical filaments of Alzheimer’s disease. Brain Pathol. 2007;17(1):83–90. doi: 10.1111/j.1750-3639.2007.00053.x 17493042 10.1111/j.1750-3639.2007.00053.x PMC8095506

[34] Hasegawa M, Watanabe S, Kondo H, Akiyama H, Mann DMA, Saito Y, et al 3R and 4R tau isoforms in paired helical filaments in Alzheimer’s disease. Acta Neuropathol. 2014;127(2):303–5. doi: 10.1007/s00401-013-1191-9 24212601 10.1007/s00401-013-1191-9 PMC3895182

[35] Sengupta A, Kabat J, Novak M, Wu Q, Grundke-Iqbal I, Iqbal K. Phosphorylation of Tau at both Thr 231 and Ser 262 is required for maximal inhibition of its binding to microtubules. Arch Biochem Biophys. 1998;357(2):299–309. doi: 10.1006/abbi.1998.0813 9735171 10.1006/abbi.1998.0813

[36] Coughlin D, Irwin DJ. Emerging Diagnostic and Therapeutic Strategies for Tauopathies. Curr Neurol Neurosci Rep. 2017;17(9):72 doi: 10.1007/s11910-017-0779-1 28785992 10.1007/s11910-017-0779-1 PMC5756477

[37] Selkoe DJ, Hardy J. The amyloid hypothesis of Alzheimer's disease at 25 years. EMBO Mol Med. 2016;8(6):595–608. doi: 10.15252/emmm.201606210 27025652 10.15252/emmm.201606210 PMC4888851

[38] Pooler AM, Polydoro M, Wegmann S, Nicholls SB, Spires-Jones TL, Hyman BT. Propagation of tau pathology in Alzheimer’s disease: identification of novel therapeutic targets. Alzheimer's Res Ther. 2013;5(5):49. 24152385 10.1186/alzrt214 PMC3978816

[39] Croft CL, Wade MA, Kurbatskaya K, Mastrandreas P, Hughes MM, Phillips EC, et al Membrane association and release of wild-type and pathological tau from organotypic brain slice cultures. Cell Death Dis. 2017;8:e2671 doi: 10.1038/cddis.2017.97 28300838 10.1038/cddis.2017.97 PMC5386587

[40] Lewis J, Dickson DW. Propagation of tau pathology: hypotheses, discoveries, and yet unresolved questions from experimental and human brain studies. Acta Neuropathol. 2016;131(1):27–48. doi: 10.1007/s00401-015-1507-z 26576562 10.1007/s00401-015-1507-z

[41] Goedert M, Spillantini MG. Propagation of Tau aggregates. Mol Brain. 2017;10(1):18 doi: 10.1186/s13041-017-0298-7 28558799 10.1186/s13041-017-0298-7 PMC5450399

[42] Golde TE. Open questions for Alzheimer’s disease immunotherapy. Alzheimer's Res Ther. 2014;6(1):3. 24393284 10.1186/alzrt233 PMC4056616

[43] Yanamandra K, Jiang H, Mahan TE, Maloney SE, Wozniak DF, Diamond MI, et al Anti-tau antibody reduces insoluble tau and decreases brain atrophy. Ann Clin Transl Neurol. 2015;2(3):278–88. doi: 10.1002/acn3.176 25815354 10.1002/acn3.176 PMC4369277

[44] Yanamandra K, Kfoury N, Jiang H, Mahan TE, Ma S, Maloney SE, et al Anti-tau antibodies that block tau aggregate seeding in vitro markedly decrease pathology and improve cognition in vivo. Neuron. 2013;80(2):402–14. doi: 10.1016/j.neuron.2013.07.046 24075978 10.1016/j.neuron.2013.07.046 PMC3924573

[45] Golde Todd E, Lewis J, McFarland NR. Anti-Tau antibodies: hitting the target. Neuron. 2013;80(2):254–6. doi: 10.1016/j.neuron.2013.10.009 24139027 10.1016/j.neuron.2013.10.009

[46] Avale ME, Rodríguez-Martín T, Gallo J-M. Trans-splicing correction of tau isoform imbalance in a mouse model of tau mis-splicing. Hum Mol Gen. 2013;22(13):2603–11. doi: 10.1093/hmg/ddt108 23459933 10.1093/hmg/ddt108 PMC3674800

